# Shear Bond Strength and Remineralisation Effect of a Casein Phosphopeptide-Amorphous Calcium Phosphate-Modified Glass Ionomer Cement on Artificial “Caries-Affected” Dentine

**DOI:** 10.3390/ijms18081723

**Published:** 2017-08-07

**Authors:** Irene Shuping Zhao, May Lei Mei, Zhuo Long Zhou, Michael Francis Burrow, Edward Chin-Man Lo, Chun-Hung Chu

**Affiliations:** 1 Faculty of Dentistry, The University of Hong Kong, Hong Kong, China; zhao110@hku.hk (I.S.Z.); mei1123@hku.hk (M.L.M.); mfburrow@unimelb.edu.au (M.F.B.); hrdplcm@hku.hk (E.C.-M.L.); 2 Department of Mechanical Engineering, The University of Hong Kong, Hong Kong, China; zhouzhuolong@gmail.com

**Keywords:** casein phosphopeptide-amorphous calcium phosphate (CPP-ACP), shear bond strength, remineralisation, glass ionomer

## Abstract

This study investigated the effect of casein phosphopeptide-amorphous calcium phosphate (CPP-ACP)-modified glass ionomer cement (GIC) on shear bond strength (SBS) and remineralisation of artificial “caries-affected” dentine. Human dentine slices were demineralised and allocated to three groups: group 1, conventional GIC; group 2, CPP-ACP-modified GIC; and group 3, resin-modified GIC. The SBS was measured using a universal testing machine (*n* = 16 per group). Remaining samples (*n* = 8 per group) were subjected to pH-cycling for 28 days. After pH-cycling, lesion depth and micro-mechanical properties at the sample-bonding interface were investigated using micro-computed tomography (micro-CT) and nano-indentation, respectively. The SBS for groups 1 to 3 were 4.6 ± 1.5 MPa, 4.2 ± 1.1 MPa, and 5.9 ± 1.9 MPa, respectively (*p* = 0.007; group 1, group 2 < group 3). Lesion depths determined by micro-CT for groups 1 to 3 were 186 ± 8 µm, 149 ± 14 µm, and 178 ± 8 µm, respectively (*p* < 0.001; group 2 < group 1, group 3). The mean (±SD, standard deviation) nano-hardness values for groups 1 to 3 were 0.85 ± 0.22 GPa, 1.14 ± 0.21 GPa, and 0.81 ± 0.09 GPa, respectively (*p* = 0.003; group 1, group 3 < group 2). The mean (±SD) elastic moduli for groups 1 to 3 were 1.70 ± 0.33 GPa, 2.35 ± 0.44 GPa, and 1.59 ± 0.13 GPa, respectively (*p* < 0.001; group 1, group 3 < group 2). The results suggest that the incorporation of CPP-ACP into GIC does not adversely affect the adhesion to artificial caries-affected dentine. Furthermore, CPP-ACP-modified GIC is superior to conventional GIC in promoting dentine remineralisation.

## 1. Introduction

Carious dentine has been classically described as consisting of two distinct layers: an inner layer of caries-affected dentine and an outer layer of caries-infected dentine [1]. Caries-infected dentine has been considered as highly demineralised and exhibiting permanent denatured collagen fibrils with a prominent disappearance of cross-linkages [2]. Unlike caries-infected dentine, caries-affected dentine is partially demineralised, and physiologically remineralisable since its collagen fibrils still show intermolecular cross-links and obvious cross-banding patterns [3]. As the caries-affected dentine should be retained during operative treatment, it will typically be the bonding substrate.

The process of dentinal caries involves dynamic, cyclical episodes of demineralisation and remineralisation [1]. As caries is caused by the imbalance between demineralisation and remineralisation, fluoride-releasing materials are advocated to enhance the formation of the less soluble fluorapatite to resist further demineralisation [4]. The anticariogenicity of fluoride is attributed not only to its inhibition on tooth demineralisation, but also its antimicrobial effect on cariogenic bacteria [5]. Glass ionomer cements (GICs) are one of the best choices of fluoride-releasing materials, being superior to other restorative materials such as giomers and polyacid-modified resin composites (compomers) from the aspect of sustained fluoride release and recharge [6]. However, the anticariogenic effect of GICs is often limited and insufficient [7]. Hence, researchers have been investigating the incorporation of various agents into GICs to promote the anticariogenic and remineralising potential of the restorative material.

Casein phosphopeptide-amorphous calcium phosphate (CPP-ACP) is a remineralising agent that has been incorporated into a conventional GIC as a bioactive additive. Casein, mainly as calcium phosphate-stabilized micellular complexes, is the principal phosphoprotein in bovine milk [8]. CPP-ACP is a nanocomplex derived from bovine milk protein, calcium, phosphate, and casein [9]. It has been demonstrated that CPP-ACP can inhibit enamel demineralisation and stimulate the remineralisation of enamel subsurface lesions in animal and human in situ studies [10,11,12]. The anticariogenic capacity of CPP-ACP has been ascribed to the ability of casein phosphopeptide to concentrate amorphous calcium phosphate at the tooth surface, and thus the levels of calcium and phosphate are increased in plaque [9]. CPP-ACP can react with fluoride ions to form amorphous calcium fluoride phosphate (ACFP). This enhances remineralisation via the formation of fluorapatite by providing soluble calcium, phosphate, and fluoride ions [8]. Furthermore, it is believed that CPP-ACP has an antibacterial effect on plaque and can reduce the growth and adherence of cariogenic bacteria [13,14].

The incorporation of CPP-ACP into GIC filling materials has been tested with promising results in the laboratory. Studies found that the incorporation of CPP-ACP into GIC can increase its compressive strength and micro-tensile bond strength to dentine, as well as enhance the release of calcium and phosphate ions under neutral and acidic conditions [15], and reduce the demineralisation of the adjacent dentine to acid challenge [16]. It has also been shown that CPP-ACP-modified GIC can increase the resistance of enamel to acid demineralisation, without adverse effects on the mechanical properties of the GIC [17]. Moreover, CPP-ACP-modified GIC could prevent the development of secondary root caries when subjected to a multi-species cariogenic biofilm challenge in a laboratory setting [18]. Another study also found that CPP-ACP-modified GIC exhibited a higher shear bond strength (SBS) to enamel compared to that of a conventional GIC [19]. However, the adhesion of CPP-ACP-modified GIC to caries-affected/demineralised dentine is not well understood. No publication in English was found in PubMed to evaluate the remineralising capability of CPP-ACP-modified GIC to caries-affected/demineralised dentine. Therefore, the aim of this study was to investigate the effect of CPP-ACP-modified GIC on the shear bond strength (SBS) and remineralisation on demineralised (artificial caries-affected) dentine. The null hypotheses tested were (1) there is no difference in the SBS to demineralised dentine of CPP-ACP-modified GIC compared with a conventional and resin-modified GIC, and (2) there is no difference in the remineralising potential of the CPP-ACP-modified GIC compared with the other GICs.

## 2. Results

### 2.1. Shear Bond Strength Test and Failure Mode Analysis

The SBS for groups 1 to 3 were 4.6 ± 1.5 Mega Pascals (MPa), 4.2 ± 1.1 MPa, and 5.9 ± 1.9 MPa, respectively (*p* = 0.007; group 1, group 2 < group 3). Resin-modified GIC exhibited the highest SBS, which was significantly different from the conventional GIC and CPP-ACP-modified GIC. The SBS of conventional GIC and CPP-ACP-modified GIC were not statistically different, although the result for the CPP-ACP-modified GIC was slightly lower (*p* > 0.05).

**Figure 1:**
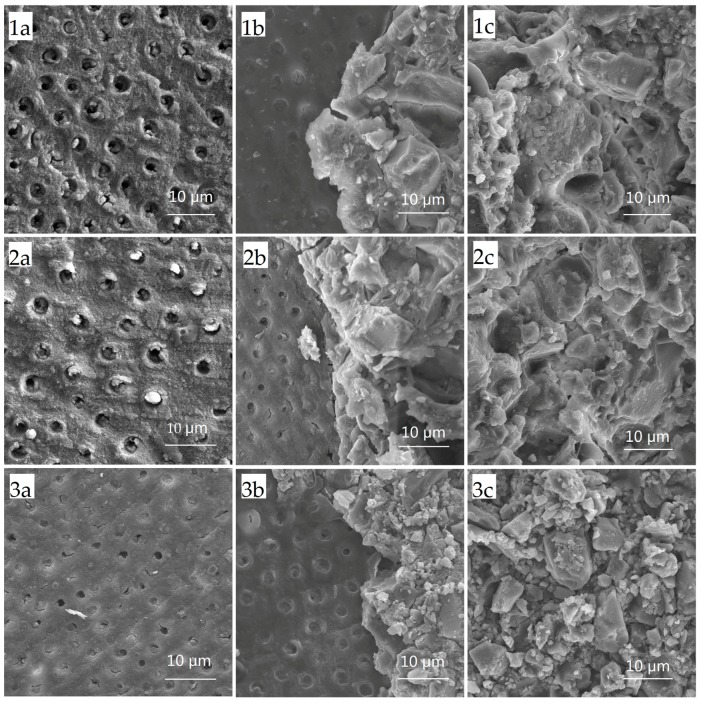
Failure modes of the dentine-GIC (glass ionomer cement) interface under scanning electronic microscope (SEM) at 1000× magnification. Adhesive failure: conventional GIC (**1a**); Casein phosphopeptide-amorphous calcium phosphate (CPP-ACP)-modified GIC (**2a**); resin-modified GIC (**3a**). Mixed failure: conventional GIC (**1b**); CPP-ACP-modified GIC (**2b**); resin-modified GIC (**3b**). Cohesive failure: conventional GIC (**1c**); CPP-ACP-modified GIC (**2c**); resin-modified GIC (**3c**).

For the failure mode, cohesive failure within the demineralised dentine layer was not observed. The distribution of fracture modes for conventional GIC, CPP-ACP-modified GIC, and resin-modified GIC were similar (*p* = 0.429) (Table 1). Mixed failure was more frequent within conventional GIC and CPP-ACP-modified GIC (*p* < 0.05). Scanning electronic microscope (SEM) images of the three failure modes are shown in Figure 1.

### 2.2. Lesion Depth from Micro-Computed Tomography (Micro-CT) Scan

**Figure 2:**
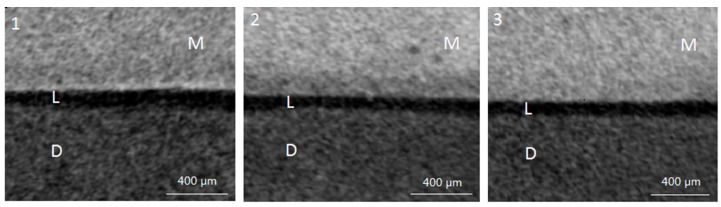
Typical micro-CT images of the three groups. (**1**) Interface of demineralised dentine and conventional GICs; (**2**) interface of demineralised dentine and CPP-ACP-modified GICs; (**3**) Interface of demineralised dentine and resin-modified GICs. M–Material, D–Dentine, L–Lesion.

One-way analysis of variance (ANOVA) found no significant difference in the baseline lesion depth among the three groups (*p* > 0.05). After pH cycling, representative pictures of micro-CT from the three groups are shown in Figure 2. The mean lesion depth (±SD) of artificial caries-affected dentine in groups 1 to 3 were 186 ± 8 µm, 149 ± 14 µm, and 178 ± 8 µm, respectively (*p* < 0.001; group 2 < group 1, group 3).

### 2.3. Nano-Hardness and Elastic Moduli from Nano-Indentation Test

**Figure 3:**
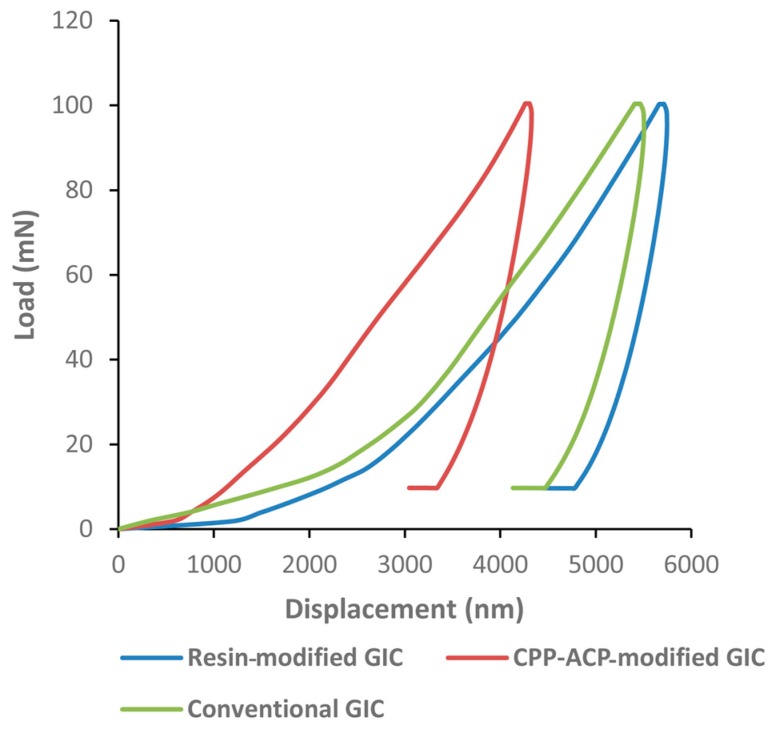
Typical force-displacement curve of the nano-indentation test on dentine. Maximum loading force: 100 mN.

The results of the nano-indentation test are shown in Table 2. Both the nano-hardness and elastic moduli were significantly higher in group 2 than those in groups 1 and 3. The typical force-displacement curve demonstrates that the nano-indenter penetrated shallower into the dentine of the samples in group 2 compared with those in groups 1 and 3 (Figure 3).

## 3. Discussion

The current work sought first to investigate whether the addition of CPP-ACP to the GIC would have any effects on the SBS to demineralised dentine and whether a difference existed in the remineralisation of the demineralised dentine. The two null hypotheses were rejected based on the results of this study. The results demonstrated that the incorporation of CPP-ACP into the GIC did not adversely affect the SBS to demineralised dentine. Furthermore, CPP-ACP-modified GIC was superior to conventional GIC and resin-modified GIC in promoting remineralisation at the demineralised dentine-GIC interface.

Fuji VII is a strontium-based glass ionomer with high fluoride release. There are very few published papers describing the adhesion of Fuji VII to tooth structure, with the most recent publications being centred on Fuji IX, also a strontium glass-based GIC from the same manufacturer. However, the powder to liquid ratio and the glass particle size of Fuji IX is slightly different from those of Fuji VII. Fuji IX is more viscous and has a higher physical strength than Fuji VII [20]. The CPP-ACP-modified GIC (Fuji VII EP) used in the current study is a recent glass ionomer cement incorporating 3% (*w/w*) CPP-ACP into Fuji VII. Regarding the bonding of CPP-ACP-modified GIC, Mazzaoui et al. [16] investigated whether the incorporation of CPP-ACP influences the micro-tensile bond strength to enamel and dentine. Nevertheless, the investigators used the conventional GIC, Fuji IX, and the concentration of incorporated CPP-ACP was 1.56% (*w/w*). GIC adhesion to caries-affected dentine has very limited data available. The work conducted by Koizumi et al. [20] is the only paper in English that evaluates the adhesion of the conventional GIC (Fuji VII) and the CPP-ACP-modified GIC to caries-affected dentine. However, their study investigated the adhesion of GICs by measuring the micro-tensile bond strength. The current study is the first one to evaluate the bonding of CPP-ACP-modified GIC to artificial caries-affected dentine by testing the SBS.

The results of this study indicate that the incorporation of CPP-ACP did not adversely affect adhesion, which is consistent with the findings of the previous study [20]. However, another paper reported that CPP-ACP-modified GIC had a significantly higher bond strength to dentine than that of conventional GIC [16]. In the current study, the highest mean SBS was found for the resin-modified GIC. This finding confirms the results of previous reports [21,22], although the actual values appear to be lower in the present study. The difference in the outcomes may be attributed to the technique, the variations in test laboratories, or even different properties of the tooth substrates. Resin-modified GIC showed a higher SBS to demineralised dentine. This may be explained by the addition of the resin, 2-hydroxyethyl methacrylate (HEMA) [22]. The demineralised dentine surface did not have a smear layer which would therefore allow the hydrophilic HEMA to easily penetrate into the exposed collagen fiber network. The addition of resin into the GIC tends to show an increased dental bonding strength which is due to the resin infiltrating the collagen fiber network to form a hybrid layer [23]. Hence, this group of GICs has both chemical and mechanical adhesion to the dentine. Cohesive failure was the most common failure mode in the bonding between GIC and dentine [24], whereas mixed failure was more frequent in this study. This difference might be ascribed to test laboratory variations. It has not yet been clearly verified whether the failure mode for a specific material can be affected by its elastic modulus or bond strength. It is known that GICs have numerous porosities that can act as stress-concentration sites where fracture may initiate. This is the most logical reason why cohesive failure is frequently observed in GICs [24].

Three conditions are crucial to remineralise partially demineralised dentine. There should be a collagen matrix serving as a scaffold for mineral crystal deposition; residual mineral seed crystallites should be present to act as growth centers; and there should be the presence of calcium and phosphate ions as mineral sources [25]. Conventional remineralisation of demineralised dentine (artificial carious) lesions usually involves the use of various concentrations of fluoride. This kind of remineralising mechanism does not depend on the nucleation of hydroxyapatite, rather it is the epitaxial growth of remnant mineral seed crystallites in the demineralised dentine [3]. Therefore, the basal part of the partially demineralised dentine, which is abundant in residual seed crystallites, can be effectively remineralised. It has been reported that the conventional GIC showed a higher fluoride ion release when compared to resin-modified GIC [26]. Although fluoride ions enhance mineral uptake, it might lead to the hypermineralisation of the lesion surface, which tends to inhibit effective remineralisation of the deeper regions of a lesion [3]. This may explain why there was no significant difference in lesion depth from the micro-CT test between conventional GIC and resin-modified GIC in this study. Micro-CT detected a significantly lower lesion depth in the group of the CPP-ACP-modified GIC compared with the other two materials. This could be due to the superior remineralising ability of the CPP-ACP-modified GIC. CPP-ACP may be considered as a calcium phosphate reservoir. It can buffer the activities of the free calcium and phosphate ions, thus helping to maintain a state of supersaturation to tooth mineral in preventing demineralisation and enhancing remineralisation [13]. This effect also influences the mechanical properties of dentine, leading to an increase in nano-hardness and elastic modulus of the demineralised dentine.

Nano-hardness is usually measured with a force of less than 700 mN. In this study, the maximum loading force was 100 mN. The mechanical properties of dentine differ according to the mineral content. Hardness is an indirect indicator of the mineral content. The results of the present study showed that dentine surfaces in the group of the CPP-ACP-modified GIC had a significantly higher nano-hardness and elastic modulus compared to the other groups. This may reflect that the CPP-ACP-modified GIC has better remineralising effects on artificial caries-affected dentine and can improve the intrinsic property of the dentine surface.

This study was conducted under controlled laboratory conditions and therefore caution should be taken when extrapolating the results to a clinical situation. In the present study, demineralised dentine was used to mimic caries-affected dentine. However, the two substrates are not exactly the same. Natural caries-affected dentine is more complex than demineralised dentine lesions in its microstructure [27]. Dentinal tubules in natural caries-affected dentine are typically occluded with acid-resistant mineral crystals, which reduce the permeability of dentinal tubules to fluid movement [3]. The two types of substrate therefore provide different conditions within dentine and will therefore result in different remineralisation efficacy. Further studies are necessary to confirm the efficacy of the CPP-ACP-modified GIC in remineralising natural caries-affected dentine.

## 4. Materials and Methods

### 4.1. Sample Preparation

**Figure 4:**
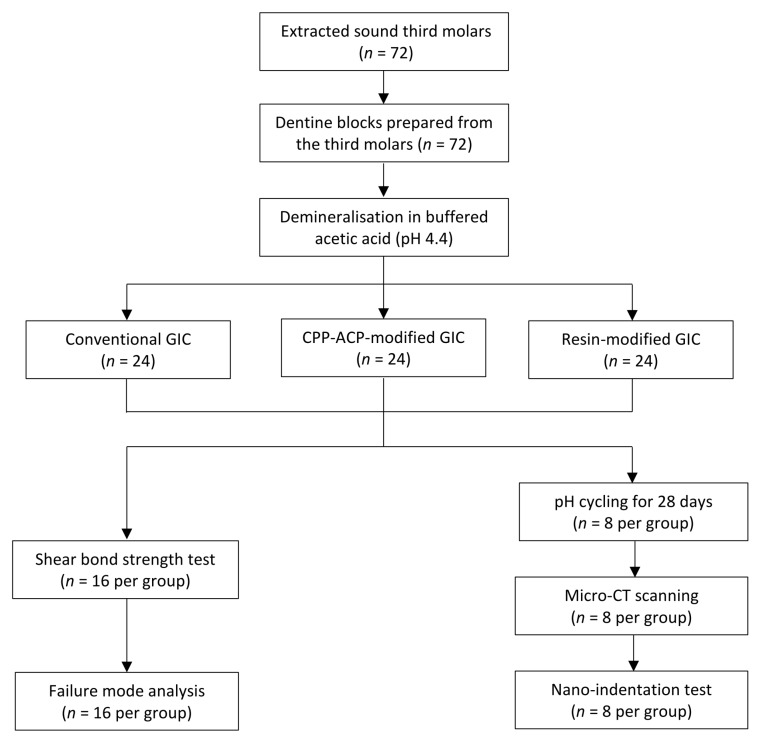
Flowchart of the study.

Seventy-two sound human third molars were obtained with patient consent under a protocol approved by the Institutional Review Board of the University of Hong Kong/Hospital Authority Hong Kong West Cluster (IRB UW12-221). Teeth were stored in 0.1% thymol solution at 4 °C before use. The protocol of this study is summarized in Figure 4.

Seventy-two 2-mm thick dentine blocks were prepared from 72 third molars by sectioning the teeth with a copper cutting disc in a hard tissue sectioner. The surfaces of the dentine blocks were polished with 4000-grit silicon carbide paper under running water. All samples were partially covered with an acid-resistant nail varnish (Clarins, Paris, France), then immersed in a demineralising solution (2.2 mM KH_2_PO_4_, 50 mM acetate, 2.2 mM CaCl_2_, pH 4.4) for seven days at room temperature [25]. A 220 ± 20-µm deep, partially demineralised baseline lesion was created on the uncoated dentine surface (confirmed by micro-CT) to mimic caries-affected dentine [3]. After lesion development, all samples were randomly allocated to three treatment groups (*n* = 24 per group). Group 1 (conventional GIC, Fuji VII, GC Corp, Tokyo, Japan), the demineralised surfaces were bonded with a conventional GIC according to the manufacturer’s instruction. Group 2 (CPP-ACP-modified GIC, Fuji VII EP, GC Corp, Tokyo, Japan), the demineralised surfaces were bonded with a GIC containing 3% *w*/*w* CPP-ACP. Group 3 (resin-modified GIC, Fuji VIII, GC Corp, Tokyo, Japan), the demineralised surfaces were bonded with auto-curing resin-modified GIC. A Teflon mold with a diameter of 3 mm and a height of 4 mm was placed on the demineralised surfaces. GIC capsules were mixed using an amalgamator (Silamat S3; Ivoclar Vivadent AG, Schaan, Liechtenstein) for 10 s. Mixed capsules were immediately removed from the mixer and the mixture was placed in the Teflon mold to form a cylindrical button. All GICs were chemically (or auto) cured. After five minutes, the Teflon mold was removed. Then, all bonded samples were stored in deionized water at 37 °C for 24 h.

### 4.2. Shear Bond Strength Test and Failure Mode Analysis

Bonded samples (*n* = 16, per group) were evaluated for the SBS using a universal testing machine (ElectroPuls 3000, Instron, Norwood, MA, USA) with a flat-edge loading head. A shear load was applied to GICs at a distance of 1.0 mm to the dentine surface. The cross-head speed was set at 0.5 mm/min. The load was recorded at failure and then the SBS was expressed in MPa by dividing the maximum shear force (Newtons) by the bonded area. The failure mode of each fractured sample was determined by a light microscope at the magnification of 20× after SBS testing. The failure patterns were categorized as three types: adhesive failure (failure between GIC and dentine), cohesive failure (failure within GIC or in dentine), and mixed failure (partial cohesive failure and partial adhesive failure) [20]. To visualize the failure mode, some of the tested samples were sputter-coated with gold and observed under an SEM (Hitachi S-4800 FEG Scanning Electron Microscope, Hitachi Ltd., Tokyo, Japan).

### 4.3. Assessment of Remineralisation

#### 4.3.1. pH Cycling

Eight bonded samples in each group underwent a pH cycling regimen at room temperature. The protocol of pH cycling followed that of Epasinghe et al. to mimic high caries risk conditions [28]. Briefly, samples were immersed in a demineralising solution (50 mM acetate, 1.5 mM CaCl_2_, 0.9 mM KH_2_PO_4_) at pH 5.0 for 16 h, followed by 8 h of immersion in remineralising solution (150 mM KCl, 20 mM 4-(2-hydroxyethyl)-1-piperazineethanesulfonic acid (HEPES), 0.9 mM KH_2_PO_4,_ 1.5 mM CaCl_2_) at pH 7.0. The pH cycling procedure was performed for 28 days. Solutions were freshly made before use.

#### 4.3.2. Micro-CT Scan

After pH cycling, samples (*n* = 8 per group) were scanned non-destructively under water to assess the lesion depth beneath materials using a SkyScan 1076 micro-CT (SkyScan, Antwerp, Belgium). A 1-mm thick aluminum filter was placed in front of the detector to eliminate low-energy radiation. Samples were scanned with a spatial resolution of 9 µm at a current of 100 µA and a voltage of 80 kV. The scan result of each sample was reconstructed by the NRecon reconstruction software (SkyScan, Antwerp, Belgium). After reconstruction, the software CTAn (SkyScan, Antwerp, Belgium) was used to view the images. Cross-sectional images exhibiting lesion area of each sample were identified from the reconstructed images. Ten images were randomly selected [25] and the lesion depth was measured using image analysis software (Image J; National Institutes of Health, Bethesda, MD, USA).

#### 4.3.3. Mechanical Analysis (Nano-Indentation Test)

Following micro-CT scanning, samples (*n* = 8 per group) were sectioned vertically for mechanical analysis in the cross-section. The nano-hardness values and elastic modulus (Young’s modulus) of the dentine adjacent to GICs was assessed by a nano-indentation test. A Berkovich diamond tip (Nano-Indenter G200, Agilent Technologies, Santa Clara, CA, USA) was used to perform the test at room temperature. The tip was calibrated using a fused-silica sample prior to evaluation. The force applied was 100 mN. Nine indentations were made on each sample, approximately 25 µm apart away from each other. The data were recorded and analyzed by Testworks 4 software (MTS Systems Corporation, Eden Prairie, MN, USA). The nano-hardness and elastic modulus of each sample were calculated via the rate-jump method [29,30], which can minimize the viscous effect of viscoelastic materials and provide reliable mechanical data during nano-indentation. The detailed information of how the results were calculated can be seen in the supplementary document. The typical force-displacement curve was presented.

### 4.4. Statistical Analysis

The data were entered into a personal computer and analyzed with IBM SPSS Version 20.0 software (IBM Corporation, Armonk, NY, USA). The normality of all data was confirmed using the Shapiro-Wilk test (*p* > 0.05). One-way ANOVA with multiple comparison (Bonferroni post hoc test) was used to compare the SBS, lesion depth, nano-hardness, and elastic moduli among the three groups. Chi-square and Fisher’s exact test were used to compare the distribution of the failure mode in the three groups. The cut-off level of significance was set as 5% for the statistical analyses.

## 5. Conclusions

The results suggest that the incorporation of CPP-ACP into GIC does not adversely affect the adhesion to artificial caries-affected dentine. Furthermore, CPP-ACP-modified GIC is superior to conventional GIC in promoting remineralisation on artificial caries-affected dentine.

## Figures and Tables

**Table 1:** Failure modes of the three groups (*n* = 16).

Group	Failure Modes (*n*)			Total (*n*)
	Cohesive	Adhesive	Mixed	
1 Conventional glass ionomer cements (GICs)	4	2	10	16
2 CPP-ACP-modified GICs	3	2	11	16
3 Resin-modified GICs	8	1	7	16

**Table 2:** Nano-hardness and elastic moduli (mean ± SD) of dentine of the three groups (*n* = 8).

Mechanical Properties	Group 1 Conventional GICs	Group 2 CPP-ACP-Modified GICs	Group 3 Resin-Modified GICs	*p* Value	Bonferroni Comparison
Nano-hardness (GPa)	0.85 ± 0.22	1.14 ± 0.21	0.81 ± 0.09	0.003	Groups 1, 3 < Group 2
Elastic moduli (GPa)	1.70 ± 0.33	2.35 ± 0.44	1.59 ± 0.13	<0.001	Groups 1, 3 < Group 2

## Supplementary Materials

Supplementary materials can be found at www.mdpi.com/1422-0067/18/8/1723/s1.

## Abbreviations

CPP-ACP	Casein phosphopeptide-amorphous calcium phosphate
SBS	Shear bond strength
GIC	Glass ionomer cement
Micro-CT	Micro-computed tomography
HEMA	2-hydroxyethyl methacrylate
SEM	Scanning electronic microscope

## References

[1] Fusayama T. (1979). Two layers of carious dentin; diagnosis and treatment. Oper. Dent..

[2] Nakajima M., Kunawarote S., Prasansuttiporn T., Tagami J. (2011). Bonding to caries-affected dentin. Jpn. Dent. Sci. Rev..

[3] Liu Y., Li N., Qi Y., Niu L.N., Elshafiy S., Mao J., Breschi L., Pashley D.H., Tay F.R. (2011). The use of sodium trimetaphosphate as a biomimetic analog of matrix phosphoproteins for remineralization of artificial caries-like dentin. Dent. Mater..

[4] Qi Y.P., Li N., Niu L.N., Primus C.M., Ling J.Q., Pashley D.H., Tay F.R. (2012). Remineralization of artificial dentinal caries lesions by biomimetically modified mineral trioxide aggregate. Acta Biomater..

[5] Featherstone J.D. (1994). Fluoride, remineralization and root caries. Am. J. Dent..

[6] Zafar M.S., Ahmed N. (2015). Therapeutic roles of fluoride released from restorative dental materials. Fluoride.

[7] Mei M.L., Zhao I.S., Ito L., Lo E.C.M., Chu C.H. (2015). Prevention of secondary caries by silver diamine fluoride. Int. Dent. J..

[8] Li J., Xie X., Wang Y., Yin W., Antoun J.S., Farella M., Mei L. (2014). Long-term remineralizing effect of casein phosphopeptide-amorphous calcium phosphate (CPP-ACP) on early caries lesions in vivo: A systematic review. J. Dent..

[9] Gupta R., Prakash V. (2011). CPP-ACP complex as a new adjunctive agent for remineralization: A review. Oral Health Prev. Dent..

[10] Reynolds E.C., Black C., Cai F., Cross K.J., Eakins D., Huq N.L., Morgan M.V., Nowicki A., Perich J.W., Riley P. (1999). Advances in enamel remineralization: Casein phosphopeptide-amorphous calcium phosphate. J. Clin. Dent..

[11] Reynolds E.C., Cain C.J., Webber F.L., Black C.L., Riley P.F., Johnson I.H., Perich J.W. (1995). Anticariogenicity of calcium phosphate complexes of tryptic casein phosphopeptides in the rat. J. Dent. Res..

[12] Reynolds E.C., Cai F., Shen P., Walker G. (2003). Retention in plaque and remineralization of enamel lesions by various forms of calcium in a mouthrinse or sugar-free chewing gum. J. Dent. Res..

[13] Reema S.D., Lahiri P.K., Roy S.S. (2014). Review of casein phosphopeptides-amorphous calcium phosphate. Chin. J. Dent. Res..

[14] Dashper S.G., Catmull D.V., Liu S.W., Myroforidis H., Zalizniak I., Palamara J.E., Huq N.L., Reynolds E.C. (2016). Casein Phosphopeptide-Amorphous Calcium Phosphate Reduces *Streptococcus mutans* Biofilm Development on Glass Ionomer Cement and Disrupts Established Biofilms. PLoS ONE.

[15] Zalizniak I., Palamara J.E., Wong R.H., Cochrane N.J., Burrow M.F., Reynolds E.C. (2013). Ion release and physical properties of CPP-ACP-modified GIC in acid solutions. J. Dent..

[16] Mazzaoui S.A., Burrow M.F., Tyas M.J., Dashper S.G., Eakins D., Reynolds E.C. (2003). Incorporation of casein phosphopeptide-amorphous calcium phosphate into a glass-ionomer cement. J. Dent. Res..

[17] Al Zraikat H., Palamara J.E., Messer H.H., Burrow M.F., Reynolds E.C. (2011). The incorporation of casein phosphopeptide–Amorphous calcium phosphate into a glass ionomer cement. Dent. Mater..

[18] Zhao I.S., Mei M.L., Burrow M.F., Lo E.C.M., Chu C.H. (2017). Prevention of secondary caries using silver diamine fluoride treatment and casein phosphopeptide-amorphous calcium phosphate modified glass-ionomer cement. J. Dent..

[19] Kucukyilmaz E., Savas S. (2016). Evaluation of shear bond strength, penetration ability, microleakage and remineralization capacity of glass ionomer-based fissure sealants. Eur. J. Paediatr. Dent..

[20] Koizumi H., Hamama H.H., Burrow M.F. (2016). Evaluation of adhesion of a CPP-ACP-modified GIC to enamel, sound dentine, and caries-affected dentine. Int. J. Adhes. Adhes..

[21] Choi K., Oshida Y., Platt J.A., Cochran M.A., Matis B.A., Yi K. (2006). Microtensile bond strength of glass ionomer cements to artificially created carious dentin. Oper. Dent..

[22] Fritz U.B., Uno S. (1996). Resin-modified glass ionomer cements: Bonding to enamel and dentin. Dent. Mater..

[23] Dursun E., Nguyen J.F., Tang M.L., Attal J.P., Sadoun M. (2016). HEMA release and degree of conversion from a resin-modified glass ionomer cement after various delays of light activation. Dent. Mater..

[24] Tanumiharja M., Burrow M.F., Tyas M.J. (2000). Microtensile bond strengths of glass ionomer (polyalkenoate) cements to dentine using four conditioners. J. Dent..

[25] Mei M.L., Ito L., Cao Y., Li Q., Lo E.C.M., Chu C.H. (2013). Inhibitory effect of silver diamine fluoride on dentine demineralisation and collagen degradation. J. Dent..

[26] Markovic D.L., Petrovic B.B., Peric T.O. (2008). Fluoride content and recharge ability of five glassionomer dental materials. BMC Oral Health.

[27] Yang B., Flaim G., Dickens S.H. (2011). Remineralization of human natural caries and artificial caries-like lesions with an experimental whisker-reinforced ART composite. Acta Biomater..

[28] Epasinghe D.J., Yiu C.K., Burrow M.F. (2015). Synergistic effect of Proanthocyanidin and CPP-ACFP on remineralization of artificial root caries. Aust. Dent. J..

[29] Tang B., Ngan A.H.W. (2012). A rate-jump method for characterization of soft tissues using nanoindentation techniques. Soft Matter.

[30] Ngan A.H.W., Tang B. (2002). Viscoelastic effects during unloading in depth-sensing indentation. J. Mater. Res..

