# Supplementary material for: Shear Bond Strength and Remineralisation Effect of a Casein Phosphopeptide-Amorphous Calcium Phosphate-Modified Glass Ionomer Cement on Artificial “Caries-Affected” Dentine
Source: Int J Mol Sci. 2017 Aug 7;18(8):1723. doi: 10.3390/ijms18081723 (PMC5578113; doi:10.3390/ijms18081723)
Supplement: Supplementary file 1 [file ijms-18-01723-s001.pdf]

Supplementary Materials

Accurate measurement of elastic modulus and hardness via a rate-jump method

In this method, the elastic modulus and hardness are evaluated at the onset of an unloading stage. Such an onset point for unloading is a rate-jump point, the true elastic modulus *Se* can be calculated from the data just before and after the unloading point using equation (1) [1],

$$\frac{1}{S_e-K}=\left(\frac{1}{S}-\frac{\dot{h_h}}{\dot{P_u}}\right)\frac{1}{\left(1-\frac{\dot{P_h}}{\dot{P_u}}\right)} \quad (1)$$

$$S_e = 2aE_r=\frac{2Ea}{1-v^2}=\frac{2Ea}{1-0.3^2}=2.2Ea \quad (2)$$

where S is the apparent tip–sample contact stiffness at the onset of unload and equals to dP/dh, P is force and h is displacement. $\dot{h_h}$ is the displacement rate just before the unload, $\dot{P_h}$ and $\dot{P_u}$ are the loading and unloading rate, respectively. *K* is the fitting parameter and equals to zero when the indentation is performed in air condition. Following the Sneddon relation [2,3] we can have equation (2), where E_r and E are the reduce modulus and the elastic modulus of the tested materials, v is the poission ratio, ($v = 0.3$ for glass-ionomer cement, [4]). a is the radius of the contact area. The contact size a can be estimated from a pre-calibrated shape function f(h_c) = π a^2 of the tip. The contact depth h_c is obtainable using the Oliver–Pharr relation [5] with the true contact stiffness *Se*, as shown in equation (3).

$$h_c = h_{max} - \varepsilon\frac{P_{max}}{S_e} = h_{max} - 0.75\frac{P_{max}}{S_e} \quad (3)$$

Where h_{max} and P_{max} are the maximum indenter displacement at the onset of unloading and the load before unloading, respectively. ε is a constant (ε =0.75 for Berkovich tip). And the hardness can be measured by equation (4),

$$H = \frac{P_{max}}{A_c} \quad (4)$$

All the raw data collected from the nanoindentor were read and calculated via Matlab R2014a software. Here we show how the results of one set data were calculated.

Sample 1:

Since samples are tested in air testing condition, K=0,

$$\frac{1}{S_e}=\left(\frac{1}{S}-\frac{\dot{h_h}}{\dot{P_u}}\right)\frac{1}{\left(1-\frac{\dot{P_h}}{\dot{P_u}}\right)} =\left(\frac{1}{17543}-\frac{257.6\times10^{-9}}{-9.993\times10^{-3}}\right)\frac{1}{\left(1-\frac{7.142\times10^{-3}}{-9.993\times10^{-3}}\right)},$$

The true elastic stiffness S_e= 2.1×10⁴ N/m;

The contact depth $h_c = h_{max} - 0.75\frac{P_{max}}{S_e} = 5700 \times 10^{-9} - 0.75 \times \frac{100\times10^{-3}}{2.1\times10^4} = 2.13 \times 10^{-6}$m

The contact size A_c =1.11× 10^{-10} m²

The contact radius a = 5.95× 10^{-6} m

The true elastic modulus $E = \frac{S_e}{2.2a}$ =1.16 × 10^9 Pa=1.16 GPa

The hardness $H = \frac{P_{max}}{A_c} = \frac{100\times10^{-3}}{1.11\times10^{-10}}= 9.01 \times 10^8$ Pa=0.9 GPa

References

1. Tang, B.; Ngan, A.H.W.; Lu, W.W. An improved method for the measurement of mechanical properties of bone by nanoindentation. *J. Mater. Sci.: Mater. Med.* **2007**, *18*, 1875–1881.
2. Johnson, K.L. Contact Mechanics; Cambridge University Press, Cambridge, UK, 1985; p. 93.
3. Sneddon, I.N. The relation between load and penetration in the axisymmetris Boussinesq problem for a punch of arbitrary profile. *Int. J. Eng. Sci.* **1965**, *3*, 47–57.

4. Akinmade, A.O.; Nicholson, J.W. Poisson's ratio of glass-polyalkenoate (“glass-ionomer”) cements determined by an ultrasonic pulse method. *J. Mater. Sci: Mater. Med.* **1995**, *6*, 483–485.
5. Oliver, W.C.; Pharr, G.M. An improved technique for determining hardness and elastic modulus using load and displacement sensing indentation experiments. *J. Mater. Res.* **1992**, *7*, 1564–1583.
